# Hypersensitivity and Allergic Reactions to PMMA-Based Acrylic Dental Materials in Patients and Dental Personnel: A Narrative Review

**DOI:** 10.3390/jcm15218580

**Published:** 2026-11-03

**Authors:** Mihaela Dana Pogan, Rahela Tabita Moca, Diana Berechet, Raluca Iurcov, Abel Emanuel Moca, Ruxandra Ilinca Matei

**Affiliations:** Department of Dentistry, Faculty of Medicine and Pharmacy, University of Oradea, 4 Universității Street, 410087 Oradea, Romania; mpogan@uoradea.ro (M.D.P.); moca.rahelatabita@didactic.uoradea.ro (R.T.M.); abelmoca@uoradea.ro (A.E.M.); rmatei@uoradea.ro (R.I.M.)

**Keywords:** polymethyl methacrylate, dental materials, contact allergy, allergic contact dermatitis, methacrylate, patch test, occupational dermatitis, dental personnel, hypersensitivity, HEMA

## Abstract

**Background/Objectives**: Polymethyl methacrylate (PMMA)-based acrylic materials are widely used in dentistry, but they are not completely biologically inert and have been associated with hypersensitivity and allergic reactions in both patients and dental personnel. This narrative review aimed to synthesize the available evidence on (meth)acrylate-related hypersensitivity reactions in dentistry, with an emphasis on clinical manifestations, immunological mechanisms, and risk factors. **Methods**: A structured literature search was conducted in PubMed, Web of Science, and Scopus (15 August–1 September 2026), following the SANRA (Scale for the Assessment of Narrative Review Articles) guideline. After screening 408 records, 17 core articles were selected and grouped into four thematic categories: prevalence and clinical relevance, molecular and immunological mechanisms, clinical manifestations in patients, and occupational sensitization in dental personnel. **Results**: Confirmed (meth)acrylate sensitization ranges from 1.4% to 8% depending on population and testing methodology, remaining lower than metal allergy but clinically relevant. Haptenization through covalent binding of electrophilic monomers to host proteins underlies sensitization, while sublethal HEMA (2-hydroxyethyl methacrylate) exposure can induce measurable cellular stress independent of overt cytotoxicity. Patient presentations range from localized contact stomatitis to acute facial swelling, whereas dental personnel, particularly dental technicians, show disproportionately high occupational incidence rates. Testing restricted to HEMA alone may miss a substantial proportion of sensitized individuals. **Conclusions**: (Meth)acrylate hypersensitivity, although relatively uncommon, warrants increased diagnostic vigilance, broader patch-test panels, and improved occupational preventive measures. The current literature remains methodologically heterogeneous, underscoring the need for standardized testing series and larger prospective studies.

## 1. Introduction

Dental materials represent a fundamental element of modern dental practice, playing an essential role in restoring the function and aesthetics of the stomatognathic system. Among these, acrylic materials, particularly polymethyl methacrylate (PMMA)-based resins, are widely used in the fabrication of dental prostheses, orthodontic appliances, and provisional restorations owing to their favorable physico-mechanical properties and low cost [1,2].

However, these materials are not completely biologically inert, being associated with various adverse effects [3]. Although these materials were introduced into dentistry as early as the first half of the 20th century and have demonstrated satisfactory clinical properties, subsequent research has highlighted certain limitations related to their biocompatibility [4,5]. The concept of biocompatibility implies a material’s ability to function within a biological environment without producing significant adverse effects on tissues [6]. In the case of acrylic materials, this condition is not always fully met, mainly due to the presence of unpolymerized components and degradation products [1,7].

Studies indicate that the main adverse effects are determined by the presence of residual monomer, particularly methyl methacrylate (MMA), as well as other compounds released from the material [5,8]. The spectrum of reported adverse reactions is broad, ranging from local manifestations such as oral mucosal irritation, contact stomatitis, and gingival inflammation, to type IV (delayed) hypersensitivity reactions, allergic contact stomatitis, and, more rarely, burning mouth syndrome [9]. In vitro studies have also revealed a cytotoxic and even genotoxic potential of MMA on cell cultures, raising further questions regarding the long-term safety of chronic exposure [10,11]. From an epidemiological standpoint, recent large-scale multicenter patch-testing data report sensitization to 2-hydroxyethyl methacrylate (2-HEMA) in approximately 2% of consecutively tested patients, with nearly one third of clinically relevant cases being of occupational origin, suggesting that the problem is not limited to patients but also concerns occupationally exposed professionals [12].

In the context of the continuous development of dental materials and increasing requirements for patient safety, analyzing the adverse effects of acrylic materials becomes essential. Understanding the underlying mechanisms and risk factors allows for the optimization of the use of these materials and the reduction in associated complications. Despite these consistent observations regarding the existence of the risk, precisely quantifying the extent and severity of adverse effects remains difficult. The current literature on this topic is highly heterogeneous: it includes numerous in vitro studies, observational studies, case series, and reviews, but very few homogeneous datasets that would allow for a robust meta-analysis of clinical effects [13,14].

An additional source of complexity lies in the diagnostic methods used to confirm hypersensitivity to acrylic dental materials. Patch testing remains the reference method for diagnosing delayed-type (type IV) contact allergy, and 2-HEMA is now included in several baseline series as a screening allergen for (meth)acrylate sensitization [12]. However, patch-test results depend heavily on methodological variables, such as the composition of the allergen series, the population tested, and the criteria used to define a positive and clinically relevant reaction. Large patch-test analyses have shown that dental material allergy cannot be adequately investigated by testing metals alone, and that (meth)acrylates should be systematically included in the test series [15]. Sensitization rates also differ considerably depending on whether symptomatic patients or occupationally exposed individuals are investigated [16], and depending on the timing of testing in relation to device placement [17]. Furthermore, the choice of screening allergens is critical: testing restricted to 2-HEMA may miss a considerable proportion of patients sensitized to other (meth)acrylates [18], although its diagnostic performance appears to vary across populations [19]. These methodological differences make it difficult to compare results across studies and may contribute to the underdiagnosis of (meth)acrylate hypersensitivity in both patients and dental personnel.

In this context, and given the methodological heterogeneity of the existing literature, the present narrative review aims to synthesize the available evidence on hypersensitivity and allergic reactions associated with PMMA-based acrylic materials used in dentistry, both in patients and in occupationally exposed dental personnel, with an emphasis on clinical manifestations, the immunological mechanisms involved, and the associated risk factors.

## 2. Materials and Methods

### 2.1. Search Strategy

A narrative review was conducted to synthesize the available evidence on hypersensitivity and allergic reactions associated with polymethyl methacrylate (PMMA)-based dental acrylic materials, both in patients and in occupationally exposed dental personnel. The review process followed the recommendations of the SANRA guideline (Scale for the Assessment of Narrative Review Articles), which provides a framework for ensuring methodological transparency, clarity, and rigor in narrative literature reviews.

The search was conducted in the electronic databases PubMed, Web of Science, and Scopus, between 15 August 2026 and 1 September 2026, using a structured Boolean search strategy, adapted syntactically for each database. The PubMed version of the search string was as follows: (methacrylate*[tiab] OR acrylate*[tiab] OR “methyl methacrylate”[tiab] OR PMMA[tiab] OR HEMA[tiab] OR EGDMA[tiab]) AND (“contact allergy”[tiab] OR “allergic contact”[tiab] OR sensitization[tiab] OR “patch test”[tiab] OR hypersensitivity[tiab] OR stomatitis[tiab]) AND (dental[tiab] OR denture*[tiab] OR prosthes*[tiab] OR orthodontic*[tiab] OR oral[tiab]).

The search was restricted to articles published within the last 5 years (2021–2026), in order to reflect the current state of clinical and epidemiological knowledge. Older, reference-standard publications were selectively included outside this structured search when they provided essential data not sufficiently covered by the recent literature (for example, the molecular mechanisms of sensitization or the basic characteristics of the monomers involved). Study selection and screening were performed by one author, and the eligibility decisions, as well as the final set of included studies, were subsequently verified by a second author against the predefined eligibility criteria. Any disagreements were resolved through discussion with a senior author.

### 2.2. Study Selection and Eligibility Criteria

The combined search across the three databases identified 408 articles (PubMed *n* = 162, Web of Science *n* = 122, Scopus *n* = 124). After removing 362 duplicates, 46 unique articles remained. Following title and abstract screening, 15 articles were excluded as irrelevant, resulting in 31 articles assessed in full text.

Inclusion criteria were original research articles, case reports, and case series; studies investigating hypersensitivity/allergic reactions to PMMA-based dental acrylic materials in patients and/or dental personnel; articles published in English.

Exclusion criteria consisted of articles focusing exclusively on other types of dental materials without a relevant acrylic component (metals, ceramics, composites without an acrylic component), studies without a documented allergic mechanism, and grey literature (theses, conference proceedings, sources not published in peer-reviewed journals).

Following full-text assessment, 15 articles were excluded, of which 10 were review articles (not suited to the aim of synthesizing primary data from original studies) and 5 for which the full text could not be identified or accessed. Thus, 16 articles met the inclusion criteria. One additional article was identified through manual search and included, resulting in a final set of 17 articles.

**Figure 1:**
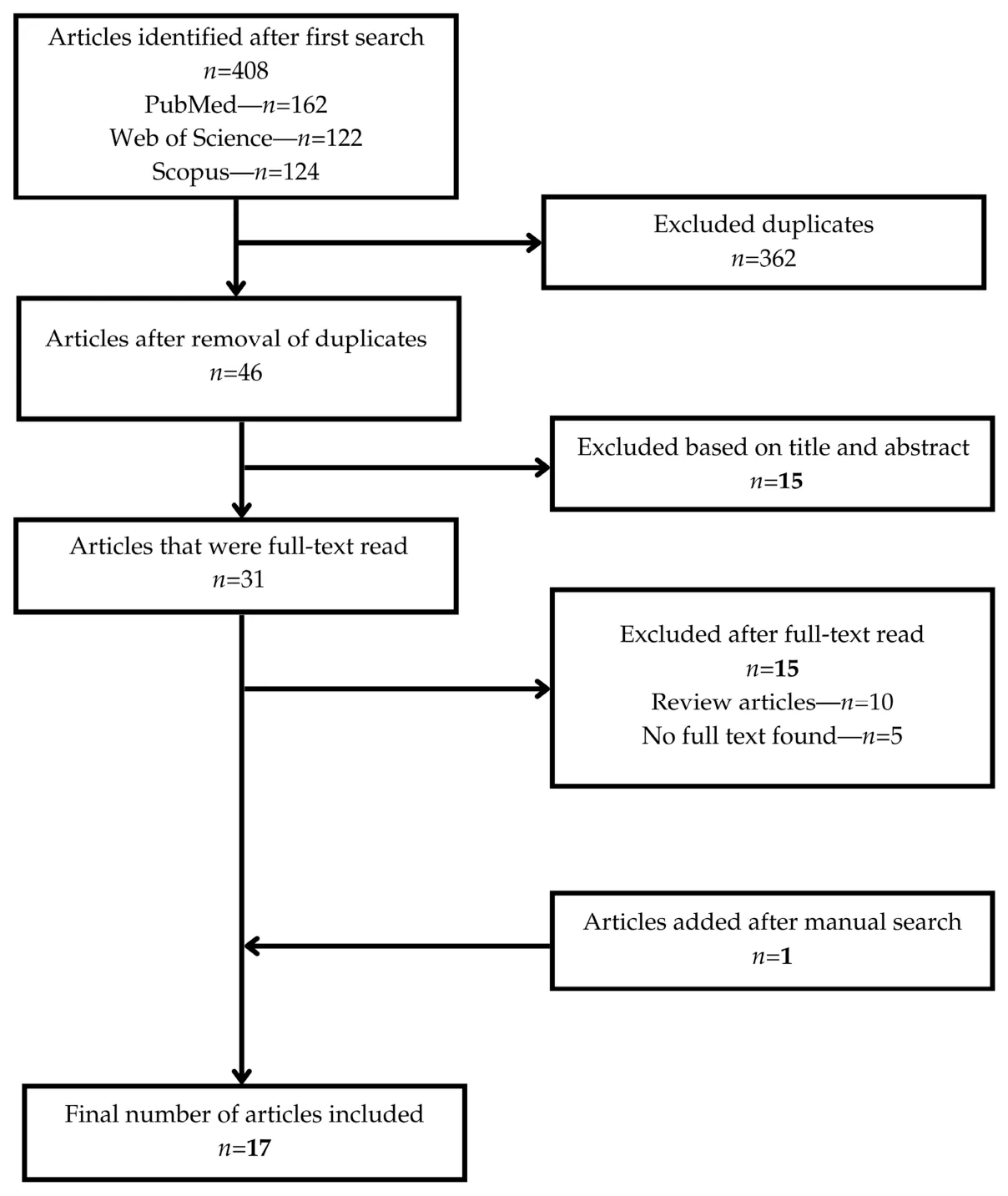
PRISMA flow diagram illustrating the study selection process.

These articles were grouped thematically and used as the basis for structuring the narrative synthesis section into four subsections: general prevalence and clinical relevance (5 articles), molecular and immunological mechanisms (1 article), clinical manifestations in patients (6 articles), and occupational sensitization/dermatitis in dental personnel (5 articles). The complete study selection flow is presented in Figure 1. The core set of 17 articles included in this narrative review is presented below:

- Forkel et al. (2024) [15];
- Olms et al. (2019) [16];
- Vallon et al. (2024) [17];
- Bizjak-Suran et al. (2026) [18];
- Romita et al. (2017) [19];
- Samuelsen et al. (2019) [20];
- Al-Gawahiri et al. (2024) [21];
- Quaade et al. (2020) [22];
- Hansel et al. (2020) [23];
- Canallatos et al. (2023) [24];
- Obayashi et al. (2023) [25];
- Aljohani et al. (2025) [26];
- Aalto-Korte et al. (2020) [27];
- Heratizadeh et al. (2018) [28];
- Aalto-Korte & Suuronen (2021) [29];
- Ryan et al. (2023) [30];
- Lyapina et al. (2016) [31].

## 3. Narrative Synthesis

### 3.1. General Prevalence and Clinical Relevance

Establishing the true prevalence of allergic reactions to dental acrylic materials represents a significant methodological challenge. Unlike other types of adverse reactions, contact allergy to (meth)acrylates is typically diagnosed through patch testing, a method that depends heavily on the allergen series chosen, the population investigated (symptomatic patients versus a general population tested preventively), and the criteria used to define a clinically relevant reaction [32,33]. Moreover, unlike allergy to metals (nickel, palladium), for which well-standardized testing series exist at the European level, there is currently no unified European series dedicated to dental materials, making direct comparison of results across studies difficult [32]. This methodological heterogeneity explains, at least in part, the considerable variability in the prevalence figures reported in the literature, a variability that becomes evident when comparing five of the most relevant recent studies on this topic [33,34].

Epidemiological data on allergic reactions to dental acrylic materials remain limited, and the reported prevalence varies considerably between studies. Forkel et al. analyzed data from the Information Network of Departments of Dermatology (IVDK) on 169,834 patients patch-tested in Germany between 2005 and 2019. Of these, only 2730 patients (1.6%) met the inclusion criteria for suspected dental material allergy (oral, labial, or perioral involvement, a dental filling material, implant, or denture as the suspected cause, and parallel testing with the baseline, dental metal, and dental technician series), the majority being women (81.2%) aged 40 years or older (92.8%). Allergic contact stomatitis was confirmed in 444 women and 68 men. Among the women, the highest sensitization rates were found for metals (nickel 28.6%, palladium 21.4%, amalgam 10.9%) and balsam of Peru (11.4%), followed by propolis (6.8%) and HEMA (4.8%, approximately 21 of 444 patients). The most relevant acrylates were HEMA, 2-hydroxypropyl methacrylate, methyl methacrylate, ethylene glycol dimethacrylate, and pentaerythritol triacrylate. Among the men, sensitization rates were highest for propolis (14.9%) and amalgam (13.6%), whereas sex-specific rates for (meth)acrylates were not reported. The authors concluded that allergic contact stomatitis to dental materials remains rare, but that patch testing should include not only metals, but also (meth)acrylates and natural substances such as propolis and balsam of Peru [15].

Similarly, Olms et al., in a cohort of 86 patients with oral symptoms at the University of Leipzig, found that allergies to methacrylate resins ranked 5th in frequency, after metals, cosmetics, composites, and medications, with more than half of patients presenting mucosal changes (contact stomatitis, lichenoid lesions). However, the authors highlighted a notable discrepancy, namely that MMA allergy ranges between 1.2 and 1.8% in patients but can reach 9.5% in occupationally exposed dental personnel [16].

In contrast to these two studies focused on symptomatic patients, Vallon et al., in 882 patients tested both pre- and post-implantation, reported a lower overall prevalence of 4.2% for acrylate sensitization (EGDMA 3.5%, HEMA 1.5%, MMA 1.4%), with no significant differences between testing timepoints, suggesting a relatively stable sensitization profile, regardless of the presence or absence of symptoms at the time of evaluation [17].

Finally, Bizjak-Suran et al., in the most recent and largest study identified (439 patients, 2016–2025), reported 6.2% positive reactions to methacrylates (HEMA 3.6%, HPMA 3.0%, EGDMA 2.5%). Unlike previous studies, they also highlighted an important methodological limitation, represented by the fact that testing restricted to HEMA would have missed 40.7% of patients with positive reactions to methacrylates, many of whom were HEMA-negative but HPMA-positive [18].

Further confirmation of this pattern comes from the study by Romita et al., who patch-tested 217 patients with symptoms suggestive of contact dermatitis at the University of Bari (2013–2014). Only 3.2% (7 patients) presented positive reactions to acrylates, the majority of which (71.4%) were cases related to dental material, confirming dentistry as the dominant source of acrylate sensitization even in a sample drawn from a general dermatology clinic [19]. Notably, 2-HEMA identified 100% of sensitized patients in this study, in contrast to the lower detection rate observed by Bizjak-Suran et al. [18], suggesting that HEMA’s performance as a screening marker varies depending on the sample and population [19].

Thus, although methodologies differ, confirmed reactions to acrylic materials remain relatively rare compared to those to metals (1.4–8%, depending on population and definition), and more recent studies converge on the idea that a panel limited to HEMA underestimates the true prevalence of sensitization [15,16,17,18,19].

### 3.2. Molecular and Immunological Mechanisms

**Figure 2:**
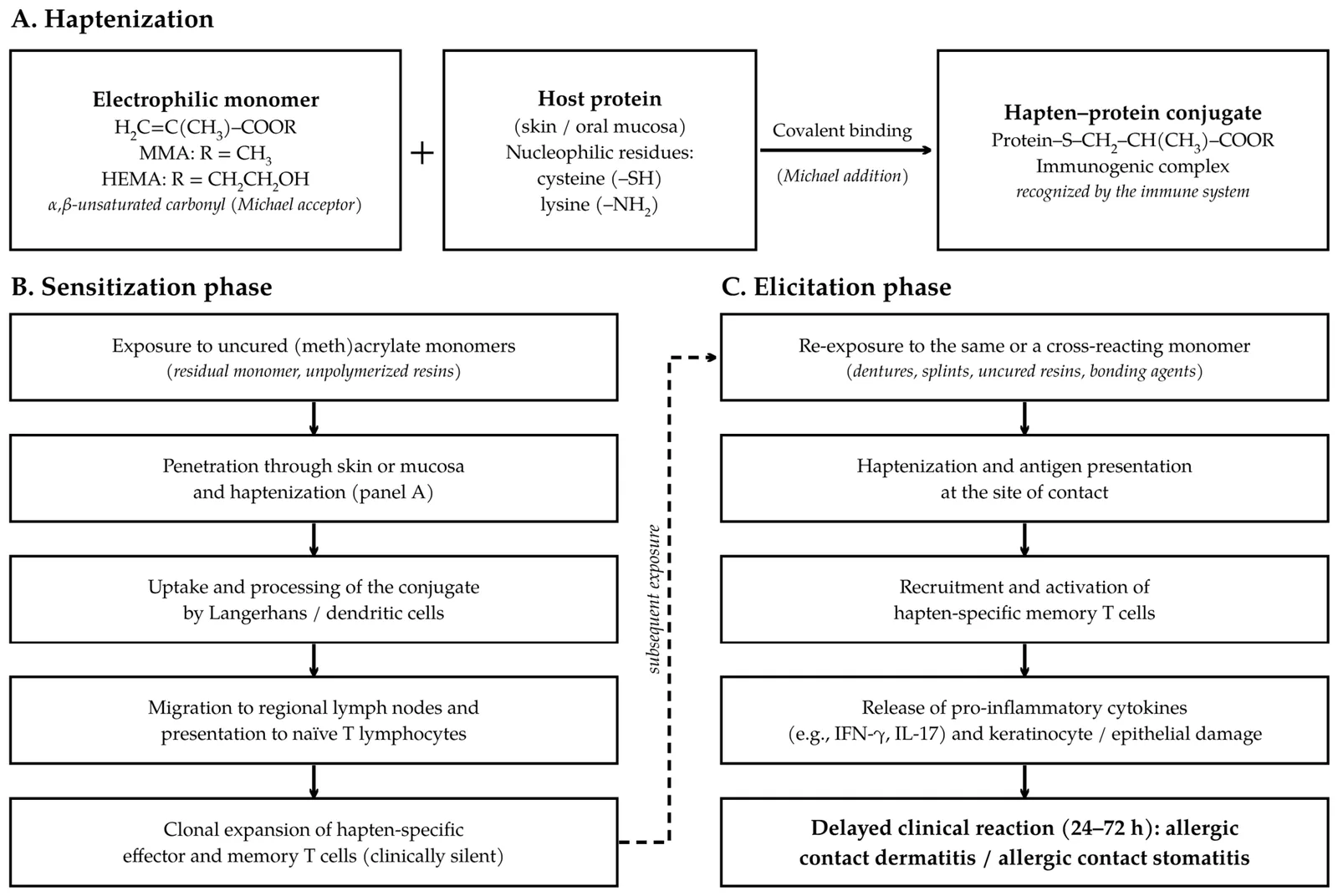
Schematic representation of the haptenization mechanism and the immunological phases of (meth)acrylate-induced allergic contact hypersensitivity.

Allergic sensitization to (meth)acrylates, although clinically documented through patch testing, has its origin at the molecular level in a relatively well-characterized mechanism. Allergic contact dermatitis represents a delayed-type (type IV) hypersensitivity reaction, mediated by T-lymphocyte-related processes, whose initial molecular event is haptenization, the covalent binding of a small molecule that is non-antigenic by itself (the hapten) to the host’s own proteins, thereby forming an immunogenic complex recognized by the immune system [35,36]. In the case of methacrylates, the electrophilic character of the monomers (such as methyl methacrylate or 2-hydroxyethyl methacrylate, HEMA) confers exactly this capacity to bind covalently to nucleophilic centers of proteins, a molecular mechanism presumed to underlie both sensitization and certain associated potential toxic effects (Figure 2) [37].

Beyond this general sensitization mechanism, a recent study by Samuelsen et al. directly investigated how HEMA, one of the most frequently used monomers in dental resins, interacts with living cells at clinically relevant exposure levels, below the threshold that produces cell death. Using an advanced proteomic technique (SILAC—stable isotope labeling with amino acids in cell culture), the authors exposed a human monocyte cell line (THP-1) to HEMA concentrations (1–4 mM) comparable to those estimated in dental pulp in vivo (0.6–3.6 mM), well below the cytotoxic concentrations used in most previous in vitro toxicity studies. The results showed that, although cell viability was not significantly affected up to 2 mM HEMA, the cells initiated a clear adaptive response to oxidative stress: exposure produced simultaneous glutathione (GSH) depletion and increased reactive oxygen species (ROS), already at the lowest concentration tested (1 mM). Proteomic analysis identified significant increases in several cytoprotective proteins, including enzymes involved in glutathione regeneration (GCLM, GSR), oxidative stress neutralization (NQO1), the pentose phosphate pathway (PGD, TKT), and molecular chaperones (HSP70), suggesting activation of the Nrf2 signaling pathway, considered the “master regulator of antioxidant responses.” The authors proposed that this activation could result either from HEMA-induced oxidative stress or from direct binding of the electrophilic monomer to the cysteine residues of the regulatory protein Keap1, the two mechanisms not being mutually exclusive [20].

A notable finding was the marked increase (nearly 30-fold) in the level of the protein pirin, associated with redox-dependent regulation of NF-κB signaling and, in other cellular contexts, with the induction of apoptosis, suggesting that, at sublethal concentrations, HEMA-exposed cells may be “primed” for alternative programmed cell death pathways, even though immediate viability is not affected. However, the authors highlight an important limitation of the study: the method used does not detect post-translational protein modifications (such as phosphorylation), and the cell line used carries a known p53 gene mutation, which limits conclusions regarding DNA damage response pathways [20].

The clinical relevance of these findings is significant. The results suggest that chronic exposure to sublethal doses of HEMA, a realistic scenario for repeatedly exposed patients and dental personnel, can induce sustained cellular stress and measurable adaptive changes, even in the absence of visible signs of cytotoxicity. This provides a possible molecular substrate for adverse effects observed clinically upon chronic exposure, beyond the simple type IV allergic reaction, and underscores the need for studies that go beyond the classical toxicity paradigm based exclusively on lethal concentrations [20].

### 3.3. Clinical Manifestations in Patients

Unlike allergic contact dermatitis in dental personnel, which is well documented in the literature [38], allergic manifestations in patients wearing or receiving acrylic dental materials are predominantly reported as isolated clinical cases, often with atypical or severe presentations, reflecting both the relative rarity of the phenomenon and the difficulty of recognizing it clinically.

Al-Gawahiri et al., in a retrospective cohort study of 360 patients (84.2% women) with intra- and perioral symptoms tested with the complete dental series, found that 285 patients (79.2%) had at least one positive patch-test reaction, but only 26 patients (7.2%) were sensitized to (meth)acrylates, most frequently to 2-HEMA (*n* = 15; 4.2%), EGDMA (*n* = 11; 3.1%), MMA (*n* = 10; 2.8%), and TEGDMA (*n* = 9; 2.5%). Overall, clinical relevance could be established in only 68 of the 208 patients (32.7%) sensitized to allergens from the dental series, highlighting that the presence of a positive patch-test reaction does not automatically imply a causal relationship with the reported symptoms. In contrast, positive reactions to (meth)acrylates were considered clinically relevant in most cases (2-HEMA 12/15, EGDMA 10/11, MMA 8/10, and TEGDMA 9/9), suggesting that, although uncommon, (meth)acrylate sensitization in symptomatic dental patients is frequently associated with the reported complaints [21].

Among individual clinical cases, a recurring pattern is allergic stomatitis associated with chronically worn acrylic dentures and dental devices. Quaade et al. reported the case of a 66-year-old woman who developed erythema, erosions, and a burning sensation of the oral mucosa after using a new occlusal splint (Orthocryl), with positive reactions confirmed to MMA and EGDMA by patch testing. Symptoms resolved completely after appropriate re-polymerization and elution of the device, illustrating the role of insufficiently eliminated residual monomer [22]. Similarly, Hansel et al. described a case of allergic contact stomatitis to an acrylic-based denture, with cross-reactivity to 2-hydroxyethyl methacrylate [23]. Canallatos et al., in a clinical report, documented a more complex case of a patient with a PMMA maxillary resection prosthesis who developed a severe allergic stomatitis reaction of the oral cavity and oropharynx. Patch testing confirmed true PMMA allergy, and the therapeutic solution consisted of replacing the prosthesis with one fabricated from urethane dimethacrylate (UDMA), an alternative material without cross-reactivity [24].

A notable and less typical clinical presentation was reported by Obayashi et al. The authors reported the case of a 55-year-old patient who developed repeated episodes of acute facial swelling following dental treatments with resin-based materials, with onset within a few hours of exposure. Testing excluded hereditary angioedema and immediate-type allergy (negative prick test), instead confirming a delayed-type hypersensitivity reaction (positive patch test) to 2-HEMA and EGDMA, a case that extends the clinical spectrum of acrylate allergy beyond classic oral manifestations (stomatitis, erosions) to facial swelling, a presentation rarely reported in the literature [25].

Beyond patients, exposure to (meth)acrylates also affects dental personnel directly, through skin contact. Aljohani et al. reported two cases of allergic contact dermatitis on the dorsal aspect of the hand in young dentists, caused by applying uncured bonding agents directly onto gloves during restorative procedures, a common habit in clinical practice, but one that allows monomer penetration through the glove material, even in the case of nitrile gloves considered more protective. Both cases resolved completely after discontinuing the habit and topical corticosteroid treatment [26].

The available cases outline a broad clinical spectrum, ranging from localized contact stomatitis and mucosal erosions to deep ulcerations and, in rare cases, acute facial swelling, all sharing as a common denominator exposure to incompletely polymerized acrylic/methacrylate monomers, either from chronically worn devices (dentures, occlusal splints) or from materials applied during treatment (cements, restorative resins). Clinical management, in all documented cases, consists of identifying and removing the source of the allergen, possibly replacing the material with cross-reaction-free alternatives (e.g., UDMA), supplemented by symptomatic treatment (topical or systemic corticosteroids, antihistamines) [21,22,23,24,25,26].

### 3.4. Occupational Sensitization and Dermatitis in Dental Personnel

Unlike patients, in whom (meth)acrylate allergy remains relatively rare, dental personnel, namely dentists, dental technicians, assistants, and hygienists, face chronic, repeated exposure to unpolymerized monomers, which is directly reflected in occupational epidemiological data. Occupational allergy to methacrylates is described in the recent literature as a “growing” phenomenon [39], with a relatively characteristic clinical pattern (facial and/or eyelid rash, eczematous fissuring of the fingertips, nail dystrophy, and periungual dermatitis) resulting from repeated, manual contact with uncured resins and adhesives. A recent scoping review confirms that dental personnel remain exposed to a wide range of chemicals with toxic potential, among which methacrylate polymers are among the most frequently documented [40]. At the molecular level, chronic exposure acquires additional relevance because residual monomers act as haptens, and repeated contact progressively increases the risk of sensitization, in contrast to the occasional or limited exposure of patients [41].

Aalto-Korte et al., analyzing 12 years of data from the Finnish Register of Occupational Diseases (2005–2016), found that dental technicians are among the professions with the highest incidence of occupational skin diseases overall (19.0 cases per 10,000 person-years, ranked 3rd after spray painters and bakers) and, more specifically, among those with the highest incidence of allergic contact dermatitis (13.57/10,000 person-years, ranked 2nd after spray painters), with acrylates identified as the primary cause in all 5 cases of ACD among dental technicians included in the analysis. This top ranking is all the more notable given that dental technicians represent a numerically small workforce, suggesting a disproportionately high occupational risk per exposed individual [27].

A similar pattern emerges from the analysis by Heratizadeh et al., based on data from the German IVDK network (2001–2015) on 226 dental technicians with occupational contact dermatitis. These individuals were diagnosed significantly more frequently with allergic contact dermatitis (37.6%) compared with the control group without occupational dermatitis (18.5%; *p* = 0.0002), and (meth)acrylates represented the most frequent cause of sensitization (67 cases), with 2-HEMA, 2-HPMA, MMA, EMA, and EGDMA being the most frequently implicated allergens. Interestingly, the sensitization and cross-reactivity spectrum has remained practically unchanged since the 1990s, suggesting a persistent occupational problem [28].

Aalto-Korte & Suuronen, in a 10-year retrospective study conducted at a specialized occupational dermatology clinic in Finland, identified 7 cases of occupational allergic contact dermatitis among dental professionals (4 assistants, 2 technicians, 1 dental hygienist) out of a total of 55 acrylic compound-related cases during the study period. Both dental technicians presented allergic reactions to methyl methacrylate (MMA), and their work products frequently also contained EGDMA, ethyl methacrylate (EMA), and urethane methacrylates, often not clearly declared on product safety data sheets. Four of the five assistants/hygienists were sensitized to 2-HEMA, a common methacrylate in dental resins [29].

Beyond “classic” contact dermatitis, the literature also documents atypical clinical patterns of occupational exposure. Ryan et al. reported a relevant case: a dentist who developed allergic contact dermatitis strictly localized to the dorsal aspect of the non-dominant hand, resulting from applying dental bonding agent directly onto the glove, a clinical habit described by the authors as the “manual tray sign” [30], the exact behavioral pattern documented by Aljohani et al. in the previous subsection, suggesting that this habit is a recurrent and recognized risk factor in the literature [26].

An additional dimension of occupational risk is co-sensitization to (meth)acrylates and other allergens frequently used in dental practice. Lyapina et al., in a study of 262 participants (dental students, dental technician students, dental professionals, and patients as a control group), investigated concomitant sensitization to methacrylic monomers and glutaraldehyde, a broad-spectrum disinfectant frequently used in dental offices. The results showed that dental professionals had a significantly increased risk of co-sensitization to TEGDMA and glutaraldehyde, while dental students and patients were identified as the groups with the highest risk of general co-sensitization to methacrylates and glutaraldehyde, with the authors attributing this increased risk among students to insufficient information and inadequate protection during the early years of clinical training [31].

Occupational data converge on a clear conclusion. Dental personnel, particularly dental technicians who directly handle unpolymerized monomers, face a significantly higher risk of (meth)acrylate sensitization than patients, with occupational incidences among the highest of all professions studied in national occupational disease registries. Recurrent risk factors include manual handling of monomers, application of uncured products directly onto gloves, insufficient training of young personnel/students, and concomitant exposure to other occupational allergens (disinfectants such as glutaraldehyde). The studies also highlight a systemic problem: product safety data sheets (SDS) do not always fully disclose the sensitizing acrylic compounds present, contributing to an underestimation of the actual exposure risk [26,27,28,29,30,31].

## 4. Discussion

The synthesis of the identified literature outlines a coherent picture of hypersensitivity reactions associated with dental acrylic materials, albeit one marked by significant methodological heterogeneity. Prevalence data, drawn from five independent studies conducted in different clinical and geographic settings, converge on the conclusion that confirmed (meth)acrylate sensitization remains relatively rare compared with metal allergy, but far from negligible, especially given the almost universal exposure of the general population to PMMA-based dental materials [15,16,17,18,19]. The central molecular mechanism of this sensitization provides a solid explanatory framework for the observed clinical manifestations [35,36,37], while recent research on HEMA interaction with cell cultures at sublethal concentrations suggests that chronic exposure, even in the absence of visible cytotoxicity, can induce sustained cellular stress with potential clinical relevance beyond the classic type IV allergic reaction [20].

From a clinical perspective, the reviewed literature highlights several important practical implications. The spectrum of manifestations in patients, from localized contact stomatitis, repeatedly described in the literature [42], to acute facial swelling [25], underscores the need for dentists and dermatologists to consider (meth)acrylate allergy even in atypical presentations. At the same time, although the present review focused on cutaneous and mucosal reactions, MMA exposure may also have a respiratory dimension. MMA has been investigated as a possible respiratory sensitizer, and although current evidence remains insufficient to firmly support this classification [43], this direction warrants further attention in chronically exposed personnel.

Several recent studies converge on a methodological conclusion with direct impact on diagnostic practice: testing limited to 2-HEMA, frequently used as a single screening allergen, may miss a substantial proportion of sensitized patients who react to other (meth)acrylates such as HPMA or EGDMA without reacting to HEMA [18]. This suggests that patch-testing protocols should include a broader panel of (meth)acrylates, rather than only the marker traditionally considered most sensitive.

For patients with confirmed PMMA allergy, the literature offers several clinically documented therapeutic solutions. Replacing the conventional acrylic material with alternatives such as urethane dimethacrylate (UDMA)-based resins has been successfully reported both in individual clinical cases [24,44] and in studies comparing the mechanical properties of hypoallergenic resins with those of conventional PMMA [45,46,47]. However, these alternatives should not be regarded as universally safe. Cross-reactivity among (meth)acrylate monomers is well recognized, and individuals sensitized to one monomer frequently react to several structurally related compounds. The cross-reactivity patterns of dental (meth)acrylates are, moreover, not mapped well enough to reliably predict a safe alternative for a given patient [48]. Concomitant patch-test reactions are particularly common between HEMA and other low-molecular-weight methacrylates such as EGDMA, TEGDMA, and MMA [49,50]. In contrast, UDMA appears to be a weak sensitizer, with few positive patch-test reactions reported even in highly exposed dental technicians [51], and a negative patch-test reaction to UDMA has been documented in a patient sensitized to HEMA and EGDMA [25]. Nevertheless, commercial UDMA-based resins are rarely composed of UDMA alone and commonly contain reactive diluent monomers (e.g., TEGDMA) and other methacrylates, which may persist as residual monomers after polymerization and may not be fully declared in product safety data sheets [29,48]. Therefore, before an alternative material is selected for a sensitized patient, patch testing should ideally include not only the individual monomers but also the specific product intended for use, in its cured form. In addition, these alternative materials remain associated with certain trade-offs regarding mechanical properties and cost, which limits their widespread adoption as a first-line solution, reserving them rather for patients with confirmed allergy [47,52].

For dental personnel, protective equipment remains a partially effective but insufficient standalone measure. Glove permeability studies consistently show that methacrylate monomers, being small molecules, penetrate most glove materials, including nitrile, considered the most protective, within intervals of only a few minutes to a few tens of minutes, especially in the presence of organic solvents [53,54]. Reported cases of hand dermatitis caused by applying bonding agent directly onto gloves [26,30] practically confirm this limitation, underscoring that effective prevention requires changing work habits (avoiding direct contact, double gloving, frequent glove changes) rather than relying exclusively on the barrier provided by the glove. These observations also have direct implications for dental education. Since (meth)acrylate sensitization is largely preventable and, once established, is usually permanent and may affect an individual’s entire professional career, awareness of this occupational hazard should be built from the earliest stages of training. Notably, dental students have been identified as a group at increased risk of co-sensitization to methacrylates and other occupational allergens, a finding attributed to insufficient information and inadequate protection during the early years of clinical training [31]. Undergraduate and postgraduate dental curricula should therefore include structured education on the sensitizing potential of uncured (meth)acrylate monomers, the limited protection offered by conventional gloves, and safe handling practices, such as no-touch techniques, avoiding the application of uncured materials onto gloves, double gloving, and frequent glove changes [26,30]. Training should also cover the early recognition of occupational hand dermatitis, the importance of timely dermatological referral and patch testing, and the critical reading of product safety data sheets, which do not always fully disclose the sensitizing components present [29]. Such educational measures, extended to dental technicians and auxiliary personnel through continuing professional development programs, could represent one of the most cost-effective strategies for reducing the burden of occupational (meth)acrylate allergy.

Beyond the strictly dental sphere, sensitizing acrylic materials also appear in other medical contexts, for example, in modern acrylic-based medical dressings and adhesives, where allergen identification is frequently hindered by manufacturers’ insufficient labeling of product composition [55]. This problem of incomplete labeling is not specific to a single field: even in the case of medical devices regulated at the European level, current labeling requirements do not always mandate explicit declaration of all sensitizing components below a certain concentration threshold [56], a regulatory gap that contributes to the underestimation and underdiagnosis of allergic reactions.

Finally, the impact of these reactions on the quality of life of affected patients and personnel should not be underestimated, as data from the broader literature on allergic contact dermatitis show a significant impairment of quality of life and workplace productivity, with absenteeism rates and impairment of daily activities comparable to those of other chronic dermatological conditions [57], a dimension often overlooked in the strictly clinical literature focused on diagnosis and treatment.

The current literature, however, presents important methodological limitations, which affect both the interpretation of existing data and the ability to draw firm conclusions. There is currently no unified European patch-testing series dedicated specifically to dental materials, unlike the well-standardized series available for metal allergy, making direct comparison of results across studies difficult and explaining, at least in part, the considerable variability in reported prevalence figures [32]. In addition, a systematic analysis of the literature on this topic showed that the vast majority of available publications are isolated case reports, with only a small number of large prospective or retrospective studies [34], a distribution that limits the generalizability of conclusions and explains why this review adopted a narrative rather than a systematic or meta-analytic approach. This heterogeneity of study design is, moreover, a problem more broadly recognized in oral health research, where published meta-analyses often include too few primary studies to allow robust estimates of statistical heterogeneity [13,14].

Based on these observations, several directions for future research appear justified. First, developing and validating a standardized European patch-testing series specifically dedicated to dental materials would be useful. Second, large prospective studies are needed to allow more precise estimates of the true prevalence of sensitization, both in patients and in dental personnel. Third, the molecular mechanisms of sublethal toxicity induced by chronic exposure, demonstrated so far in a single proteomic study [20], warrant further investigation. Finally, the development and long-term clinical validation of hypoallergenic alternative materials [45,46,47] remains an active field, with the potential to significantly reduce the burden of this problem for already sensitized patients.

Lastly, the present review has its own limitations, inherent to its narrative nature. Although the search strategy was formalized and the eligibility decisions were verified by a second author, initial screening was performed by a single author rather than by two reviewers working independently, which may introduce a degree of subjectivity in the interpretation of eligibility criteria. Furthermore, the corpus of 17 core articles analyzed, while thematically relevant, remains limited in size and does not allow for a quantitative synthesis of prevalence or risk data. These limitations are, however, consistent with the review’s own central observation, namely that the heterogeneity of the existing literature on this topic makes a robust meta-analysis difficult to achieve, at least for the time being.

## 5. Conclusions

PMMA-based acrylic materials can induce type IV hypersensitivity reactions both in patients and, to a greater extent, in chronically exposed dental personnel. Although relatively rare compared with metal allergy, these reactions are not negligible, and their varied clinical spectrum justifies increased diagnostic vigilance.

Testing limited to 2-HEMA remains insufficient, and a broader panel of (meth)acrylates should be systematically integrated into diagnostic protocols. For dental personnel, effective prevention requires changing habits related to handling uncured materials rather than relying exclusively on protective equipment. To this end, education on the risks of (meth)acrylate exposure and on preventive measures should be integrated into undergraduate dental curricula and continuing professional development programs for dentists, dental technicians, and auxiliary personnel.

The current literature remains too heterogeneous and predominantly based on case reports to allow a robust quantitative synthesis, and the absence of a standardized European series dedicated to dental materials represents an important gap for future research.

## Data Availability

This article is a narrative review and does not report any new data. All data supporting the findings discussed are available from the cited literature.

## Abbreviations

The following abbreviations are used in this manuscript:

ACD	Allergic contact dermatitis
EGDMA	Ethylene glycol dimethacrylate
EMA	Ethyl methacrylate
GCLM	Glutamate–cysteine ligase modifier subunit
GSH	Glutathione
GSR	Glutathione reductase
HEMA, 2-HEMA	2-Hydroxyethyl methacrylate
HPMA, 2-HPMA	2-Hydroxypropyl methacrylate
HSP70	Heat shock protein 70
IFN-γ	Interferon gamma
IL-17	Interleukin 17
IVDK	Information Network of Departments of Dermatology
Keap1	Kelch-like ECH-associated protein 1
MMA	Methyl methacrylate
NF-κB	Nuclear factor kappa B
NQO1	NAD(P)H quinone dehydrogenase 1
Nrf2	Nuclear factor erythroid 2–related factor 2
PGD	6-Phosphogluconate dehydrogenase
PMMA	Polymethyl methacrylate
PRISMA	Preferred Reporting Items for Systematic Reviews and Meta-Analyses
ROS	Reactive oxygen species
SANRA	Scale for the Assessment of Narrative Review Articles
SDS	Safety data sheet
SILAC	Stable isotope labeling with amino acids in cell culture
TEGDMA	Triethylene glycol dimethacrylate
UDMA	Urethane dimethacrylate
TKT	Transketolase
